# A polymorphism site in the pre-miR-34a coding region reduces miR-34a expression and promotes osteosarcoma cell proliferation and migration

**DOI:** 10.3892/mmr.2014.2582

**Published:** 2014-09-18

**Authors:** HONGLIN LV, JINGFANG PEI, HONGTAO LIU, HAIYAN WANG, JUN LIU

**Affiliations:** 1 Department of Spinal Orthopedics, Medical College of Qingdao University, Yantai, Shandong 264000, P.R. China; 2 Central Laboratory, Yantai Yuhuangding Hospital, Medical College of Qingdao University, Yantai, Shandong 264000, P.R. China; 3 Department of Spinal Orthopedics, Wendeng Orthopedics Hospital, Wendeng, Weihai, Shandong 26440, P.R. China

**Keywords:** osteosarcoma, microRNA, pre-miRNA, polymorphism, cell proliferation

## Abstract

Osteosarcoma (OS) is the most prevalent primary malignant bone tumor in children and young adults, its complex etiology involving a combination of environmental and genetic factors. MicroRNA (miRNA) is a short, non-coding regulatory RNA molecule that represses gene expression by imperfectly base-pairing to the 3′ untranslated region of target mRNAs. Evidence has shown that alterations in the expression of miRNA are involved in the initiation, progression, and metastasis of human cancers. It is believed that miRNAs function both as tumor suppressors and oncogenes during cancer development. In the present study, three tumor-associated miRNAs (miR-21, miR-34a and miR-146a) coding regions were screened in Chinese-Han OS patients. A G>A variation in the pre-miR-34a coding region was found to be associated with higher OS morbidity. By detecting the mature miR-34a expression in cells transfected with pre-miR-34a expression vectors of different genotypes using quantitative polymerase chain reaction, it was demonstrated that the G>A variation reduced miR-34a expression *in vitro.* This was in accordance with the data collected from tumor tissue and patient serum samples. Subsequently, a dual-luciferase reporter assay and western blot analysis were used to detect the site variation effect on the expression of c-Met, a target gene of miR-34a. The G>A variation downregulated the suppression of c-Met in two OS cell lines. Furthermore, it was found that reduced miR-34a expression decreased the suppression of OS cell proliferation *in vitro*. In conclusion, the present study established the association between miR-34a and the risk of suffering OS in a Chinese Han population by identifying one functional single nucleotide polymorphism site in pre-miR-34a. These findings may give insight into the mechanism of OS development and create an opportunity to approach the diagnosis and treatment of OS.

## Introduction

Osteosarcoma (OS), the eighth most common form of childhood cancer, derives from primitive bone forming mesenchymal cells and is the most prevalent primary bone malignancy. The complex etiology involves a combination of environmental factors and genetic impairments. OS has a bimodal age distribution, with the first peak during adolescence and the second peak in later adulthood. The first peak occurs in 10–14-year-olds, coinciding with the pubertal growth spurt which suggests a close correlation between the adolescent growth spurt and OS (1). Since the development of medical technology, the five-year survival rate of patients carrying OS has been significantly improved (2), while the cure rate of patients carrying OS remains poor (3).

MicroRNA (miRNA) is a short non-coding regulatory RNA that represses gene expression by imperfectly base pairing to the 3′ untranslated region (3′UTR) of target mRNAs. Evidence has shown that alterations in the expression of miRNA are involved in the initiation, progression, and metastasis of human cancer. It is believed that miRNAs can function as tumor suppressors as well as oncogenes during cancer development (4,5). Studies have indicated that miRNAs have an important role in OS pathogenesis and progression (6–8).

In the present study, in order to gain insight into the role of miR polymorphism in OS, the coding regions of three miRNAs (miR-21, miR-34a and miR-146a) were screened in 103 patients; these miRNAs are associated with numerous types of cancer pathogenesis (9–11). The effect of variation in pre-miR-34a coding regions on miR-34a expression and OS cell proliferation were investigated *in vitro* and compared with data from tissue and blood serum samples of patients with OS were analyzed. Furthermore, the effect of site variation on the expression of the c-Met oncogene, a target gene of miR-34a, was investigated using western blot analysis and a luciferase reporter assay.

## Materials and methods

### Study population and tissue samples

A total of 65 pairs of surgically resected OS (prior to neoadjuvant chemotherapy administration) and adjacent normal bone tissue were acquired from Yantai Yuhuangding Hospital (Qingdao University, Shandong, China) between January 2010 and June 2012. Written informed consent was obtained from all patients.

The peripheral blood samples of 103 OS patients were also obtained from Yantai Yuhuangding Hospital. The control group consisted of samples from 201 Han-Chinese individuals and were also collected from Yantai Yuhuangding Hospital. The present study was approved by the Ethics Committee of Yantai Yuhangding Hospital (Yantai, China).

### DNA collection and genotyping

DNA from the adjacent normal tissues and tumor tissues of the OS cancer cohort were isolated by using the TIANamp Genomic DNA kit (Tiangen, Beijing, China). DNA from blood samples was extracted using the TIANamp Blood DNA kit (Tiangen).

DNA specimens were amplified using standard polymerase chain reaction (PCR) protocols. The PCR products were sequenced in the forward direction with the ABI 3730xl sequencing platform (Applied Biosystems, Foster City, CA, USA). The sequencing results were analyzed by using DNAMAN version 5.2.9 (Lynnon Corporation, Quebec, Canada) and Chromas Lite software version 2/22 (Technelysium Pty, Ltd., Shannon Ireland). The PCR primers used for miR-34a sequencing were 5′-CCCACATTTCCTTCTTATCAACAG-3′ and 5′-GGCATCTCTCGCTTCATCTT-3′.

### Quantitative polymerase chain reaction (qPCR)

qPCR analysis was used to determine the relative expression levels of miR-34a-5p. Total RNA was extracted from tissues and cells using TRIzol (Invitrogen Life Technologies, Carlsbad, CA, USA) according to the manufacturer’s instructions. The expression levels of miR-34a-5p were detected using TaqMan miRNA RT-Real Time PCR. Single-stranded complementary DNA (cDNA) was synthesized by using TaqMan MicroRNA Reverse Transcription kit (Applied Biosystems) and then amplified using TaqMan Universal PCR Master Mix (Applied Biosystems) together with miRNA-specific TaqMan dihydrocyclopyrroloindole tripeptide minor groove binder probe: miR-34a-5p (Applied Biosystems). The U6 small nuclear RNA (snRNA) was used for normalization. Each sample in each group was measured in triplicate and the experiment was repeated three or more times for the detection of miR-34a-5p. Results are expressed as the mean ± standard error of the mean.

### Secondary structure prediction

The secondary structure of a 110-base pair (bp) pre-miR-34a sequence including mutation site was predicted using the RNA fold web server (http://rna.tbi.univie.ac.at/cgi-bin/RNAfold.cgi).

### MiR-34a expression vectors

To construct mir-34a expression vectors, fragments (533 nt) corresponding to pre-mir-34a and its flanking regions (previously determined to have the two genotypes) were amplified from cDNA and cloned into the pcDNA3.1 vector (Invitrogen Life Technologies). The sequences of these two vectors were confirmed by direct sequencing; the only difference was in the mutation site. The primers were miR-34a-F/XhoI 5′-CCGCTCGAGGTCACCATGCCTGGCTAATTGAGGAGG-3′ and mir-34a-R/XbaI 5′-GCTCTAGAACTATTCTCCCTACGTGCAAAC-3′.

### Dual luciferase assay

The full length of the 2262-bp c-MET 3′UTR were cloned downstream of the firefly luciferase coding region in the pmirGLO vector (Promega, Madison, WI, USA) to generate the luciferase reporter vector. For luciferase reporter assays, SAOS-2 and U2OS cells, obtained from the Cell Resource Center of Peking Union Medical College (Beijing, China), were seeded in 48-well plates at a density of 1×10^4^. miR-34a expression and luciferase reporter vectors were co-transfected by using Lipofectamine 2000 (Invitrogen Life Technologies). Two days later, cells were harvested and assayed with the Dual-Luciferase Reporter Assay system (Promega). Each treatment was performed in triplicate in three independent experiments. The results were expressed as relative luciferase activity (Firefly LUC/Renilla LUC).

### MTT cell proliferation assay

The proliferation capacity of cells was evaluated using the MTT assay, performed according to the manufacturer’s instructions (Sigma, St. Louis, MO, USA), in 96-well plates. In brief, cells were seeded at a density of 2,000 cells per well containing 100 μl culture medium and cultured overnight. Every 24 h interval, 20 μl 5 mg/ml MTT reagent was added to each well and cells were incubated for a further 4 h at 37°C. The medium was then removed, and 100 μl dimethyl sulfoxide (DMSO; Sigma) was added to each well to dissolve the formazan. Optical density (OD) was evaluated by measuring the absorbance at a test wavelength of 490 nm and a reference wavelength of 630 nm. Wells without cells (DMSO alone) were used as blanks. Each group contained six wells; experiments were repeated three times independently and the results are expressed as the mean ± standard deviation.

### Western blot analysis

Protein extracts were boiled in an SDS/β-mercaptoethanol sample buffer (Sigma), and 30 μg sample was loaded into each lane of 8% polyacrylamide gels. The proteins were separated by electrophoresis, and the proteins in the gels were blotted onto polyvinylidene difluoride membranes (Amersham Pharmacia Biotech, St. Albans, UK) by electrophoretic transfer. The membrane was incubated with rabbit anti-c-Met polyclonal antibody (1:5,000; Abcam, Cambridge, MA, USA) and/or mouse anti-β-actin monoclonal antibody (1:5,000; Santa Cruz Biotechnology Inc., Santa Cruz, CA, USA) for 1 h at 37°C. The specific protein antibody complex was detected using horseradish peroxidase-conjugated goat anti-rabbit and rabbit anti-mouse immunoglobulin G (1:5,000; Santa Cruz Biotechnology, Inc.). Detection by the chemiluminescence reaction was carried using the enhanced chemiluminescence kit (Pierce, Appleton, WI, USA). The β-actin signal was used as a loading control.

### Detection of serum c-Met concentration using ELISA

Serum c-Met levels were detected using the sandwich ELISA method with rabbit and mouse anti-c-Met antibodies (Abcam). The relative concentrations were compared using OD values directly. The results were analyzed using the Mann-Whitney U test. P<0.05 was considered to indicate a statistically significant difference between values.

### Statistical analysis

Data were analyzed by using SPSS Statistical Package version 16 (International Business Machines, Armonk, NY, USA). Analysis of two independent groups were performed using a Student’s t-test. Results of tissue miR-34a levels were analyzed using the Mann-Whitney U test. P<0.05 was considered to indicate a statistically significant difference.

## Results

### Genotypes and risk of OS

Coding regions (pre-miR-146a, -21 and -34a) were scanned in 103 OS patients and 201 healthy controls in order to investigate the correlation between nucleotide variants of these three candidate miRNA coding regions and the pathogenesis of OS. Although no sequence changes had previously been described, the present study identified that the rare allele A of rs72631823 was highly correlated to OS (OR=15.65, 95% CI=[5.41, 45.29]) (Table I).

### The G to A variation can predict pri-miR-34a stability and reduce mature miR-34a expression

**Figure 1:**
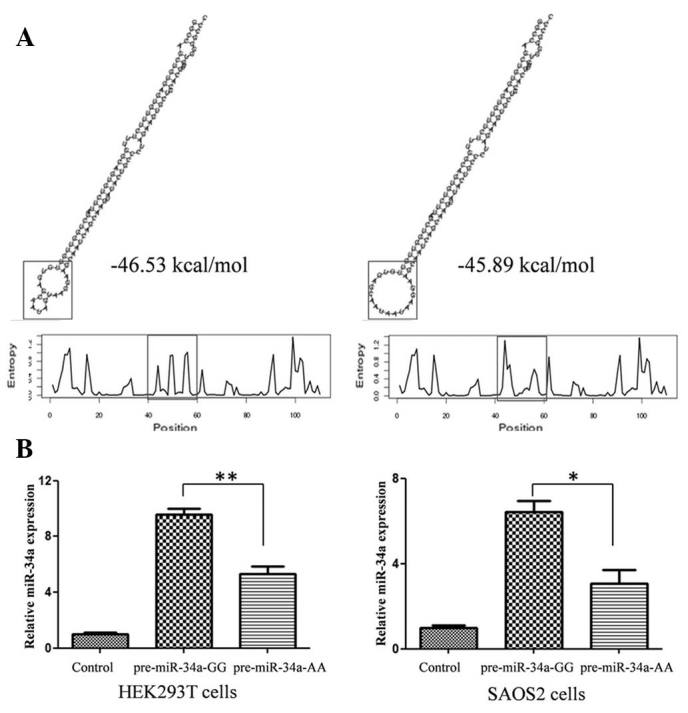
Nucleotide variation altered the predicted secondary structure of pre-miR-34a and reduced mature miR-34a expression. (A) Predicted structure of 110 nt pre-miR-34a. Single nucleotide replacement by A caused changes in the size of one loop, one stem structure and an increase in entropy. (B) miR-34a-5p expression levels generated by different genotypes of pre-miR-34a were detected using quantitative polymerase chain reaction. miR levels were normalized against U6 small nuclear RNA. The Y-axis displays the relative expression of miR-34a normalized to U6. Results are expressed as the mean±standard error of the mean. ^*^P<0.05, ^**^P<0.01. miR, micro RNA.

**Figure 2:**
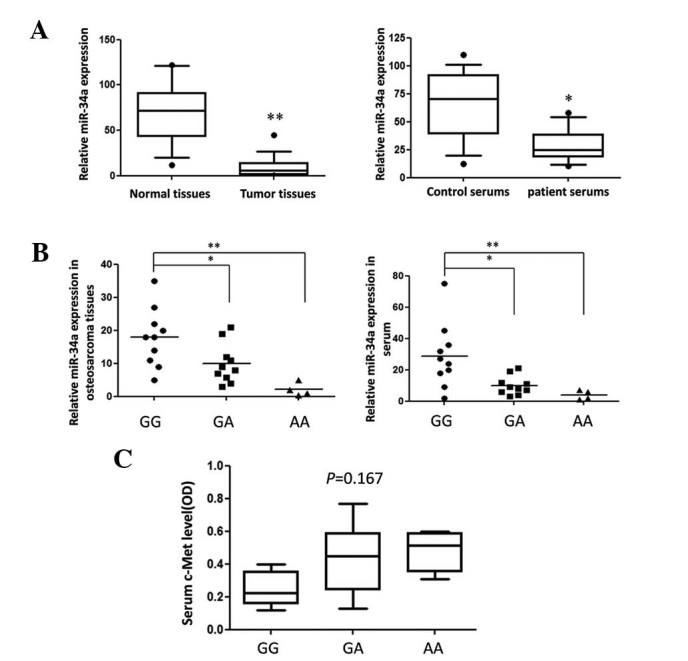
Relative miR-34a expression in OS tissues. The expression levels of miR-34a-5p in (A) 60 random individual samples and (B) 24 genotyped samples of OS tissue were detected using quantitative polymerase chain reaction. Statistical analyses were performed to evaluate the overall trend of miR-34a in all OS tissues and adjacent normal bone tissues. miR levels were normalized against U6 small nuclear RNA. The Y-axis displays the relative ratio of miR-34a normalized to U6. ^*^P<0.05, ^**^P<0.01. (C) The serum c-Met concentration in different genotypes of OS patients was measured using ELISA. The results were analyzed by the Mann-Whitney U test. P<0.05 was considered statistically significant. Results are expressed as the mean±standard deviation. miR, micro RNA; OS, osteosarcoma.

To further explore the function of the mutation site, the predicted secondary structures of two pri-miR-34a genotype molecules were compared. As shown in Fig. 1A, rare allele A caused an apparent change in loop size and a higher than predicted ΔG from −46.53 kcal/mol to −45.89 kcal/mol. The expression of mature miR-34a-5p generated by different miR-34a-5p genotype expression vectors in two different cell lines was detected using qPCR. The G to A variation caused an ~50% reduction in mature miR-34a-5p expression (Fig. 1B), the result of which correlated with data from OS tissues (Fig. 2B).

### Disturbed miR-34a expression weakens the suppression effect on c-Met expression

**Figure 3:**
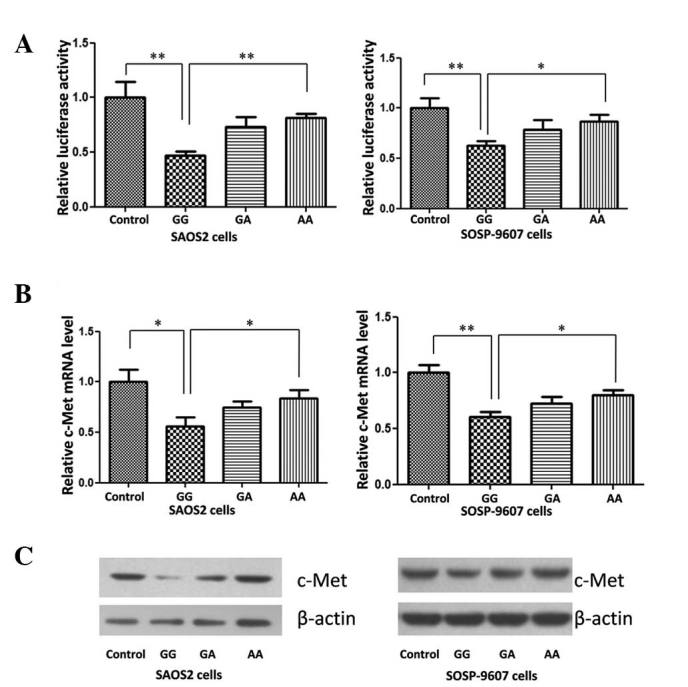
G>A variation weakens the suppression of c-Met, reduces miR-34a expression and upregulates c-Met expression in transiently transfected OS cells. (A) The relative luciferase activity of the reporter vector with the 3′-UTR of c-Met was detected in the presence of two genotypes pre-miR-34a. The G>A variation decreased the suppression of c-Met by 63.2% in SAOS2 cells and 23.9% in SOSP-9607 cells. (B) Quantitative polymerase chain reaction was used to detect mRNA levels of c-Met. Results are expressed as the mean±standard deviation. (C) Western blot analysis was used to detect c-Met expression in transfected genotypes miR-34a. ^*^P<0.05, ^**^P<0.01. miR, micro RNA; OS, osteosarcoma; 3′UTR, 3′ untranslated region.

Studies have reported that miR-34a directly repressed the expression of c-Met in HeLa cells (12), suppressed brain tumor growth through targeting c-Met (13) and acted as a tumor suppressor in uveal melanoma cell proliferation and migration through the downregulation of c-Met (14). Yan *et al* (15) confirmed that miR-34a repressed OS cell proliferation and migration *in vitro* and *in vivo*. In order to detect the impact of G>A variation on miR-34a target genes in the present study, a c-Met reporter system was constructed. The results of the dual luciferase assay indicated that in the two OS cell lines, expression of c-Met was significantly downregulated following transfection with different miR-34a genotype expression vectors, compared with that of normal control cells (Fig. 3A).

### Detection of c-Met expression using qPCR and western blot analysis

As shown in Fig. 3B and C, the expression of c-Met in two OS cell lines was significantly repressed by the miR-34 GG genotype expression plasmid. A>G variation reduced the repressive effect of miR-34a, 48 h post-plasmid transfection.

### Disturbed miR-34a expression weakens the suppression of OS cell proliferation in vitro

**Figure 4:**
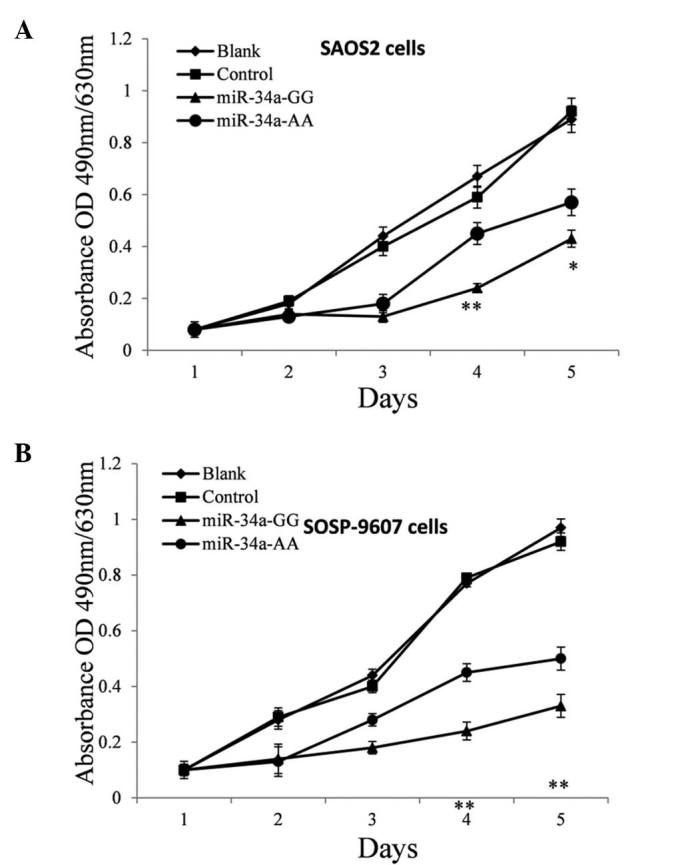
G>A variation weakens the suppression of osteosarcoma cell line proliferation. (A) SAOS2 and (B) SOSP-9607 cells were transfected with pcDNA3.1 and miR-34a expression vectors of different genotypes. Cell proliferation was determined by MTT assays every 24 h following transfection. All experiments were performed a minimum of three times. Differences between miR-34a-GG and mir-34a-AA were analyzed using Studen’s t-test (^*^P<0.05 and ^**^P<0.01). Results are expressed as the mean±standard deviation. miR, micro RNA; pcDNA, plasmid cytomegalovirus promoter DNA; OD, optical density.

Based on current knowledge of the function of c-Met, it was predicted that the reduction of miR-34a expression should promote cell proliferation (16–17). Therefore, a proliferation assay was performed on SAOS2 and SOSP-9607 cells treated with pre-miR-34a of different genotypes in order to investigate the effect of polymorphism on the anti-tumor efficacy of miR-34a in OS cells. The MTT assay was performed every 24 h post plasmid transfection. As hypothesized, these pre-miR-34a GG genotypes suppressed the proliferation of SAOS2 (Fig. 4A) and SOSP-9607 (Fig. 4B) cells significantly, most notably on the fourth day post-transfection (P=0.0015 and 0.0047) and the A>G mutation reduced the suppressive effect by nearly 20% (P=0.033) and 35% (P=0.0031), respectively.

## Discussion

Recent evidence has demonstrated that altered miRNA expression correlates with various human diseases, particularly numerous types of cancer. The behavior of miRNAs is complex as they regulate hundreds of targets, resulting in the downregulation of numerous target genes, including oncogenes and tumor suppressors. Therefore, exploring their clinical potential is particularly promising for the identification of novel diagnosis and treatment methods for patients with cancer.

In mammalian cells, following transcription by RNA polymerase II, primary miRNA (pri-miRNA) is processed by Drosha and converted into an ~70 nt hairpin precursor miRNA (pre-miRNA). Through interactions with exportin-5 and Ras-related nuclear protein-guanosine triphosphate, pre-miRNA is transported into the cytoplasm, where it is further processed by RNA polymerase III and Dicer prior to finally being turned into mature miRNA of ~22 nt (18). Increasing evidence indicated that nucleotide variation in miRNA sequence can alter miRNA expression and/or maturation and therefore be involved in the occurrence of diseases (19,20). In the present study, three tumor-associated miRNA coding regions were scanned in Chinese-Han OS patients in order to investigate the genetic predisposition of OS. It was found that a G>A variation in the pre-miR-34a coding region was associated with higher morbidity in patients with OS. The expression of mature miR-34a in cells transfected with pre-miR-34a expression vectors of different genotypes was detected using qPCR. It was found that the G>A variation reduced miR-34a expression *in vitro,* which correlated with the data from tumor tissue and patient serum samples. The function of an miRNA is mainly dependent on which genes are suppressed by this miRNA. Therefore, in order to investigate the biological function of G>A variation, a dual-luciferase assay and western blot were used to detect the site variation effect on c-Met expression, a target gene of miR-34a. As hypothesized, G>A variation weakened the suppression of c-Met in the two OS cell lines. However, the serum c-Met concentration in patients of different genotypes was detected and no significant differences were found. This may be due to the fact that miRNA is only one of the numerous factors that contribute to the regulation of gene expression.

In conclusion, the present study established a correlation between miR-34a and the risk of OS in a Chinese Han population by identifying one functional single nucleotide polymorphism site in pre-miR-34a. These findings may give insight into the mechanisms of OS development and create an opportunity to approach the diagnosis and treatment of OS.

## Figures and Tables

**Table I:** Genotype frequencies of rs72631823 in patients and controls and their association with osteosarcoma.

Genotype	Patients (n=103), freq	Controls (n=201), freq	OR (95% CI)	P-value
rs72631823 (G>A)				
A	28 (0.14)	4 (0.010)	15.65 (5.41, 45.29)	<0.001
G	178 (0.86)	398 (0.99)	0.064 (0.022, 0.18)	
A A	4 (0.04)	0 (0.00)		
A G	20 (0.19)	4 (0.020)	11.87 (3.94, 35.79)	<0.001
G G	79 (0.77)	197 (0.98)	0.067 (0.023, 0.20)	
rs2910164 (G>C)				
C	27 (0.131)	72 (0.179)	0.69 (0.43, 1.12)	0.13
G	179 (0.869)	330 (0.821)	1.45 (0.90, 2.33)	
C C	3 (0.078)	4 (0.020)	1.48 (0.32, 6.73)	0.10
C G	21 (0.48)	64 (0.318)	1.68 (0.98, 2.89)	
G G	79 (0.45)	133 (0.662)	0.55 (0.31, 0.96)	

OR, odds ratio; OR 95% CI, 95% confidence interval; freq, frequency.
